# Clinical profile of early-onset Alzheimer’s disease in Peru: case series from a neurological care center

**DOI:** 10.17843/rpmesp.2025.422.14413

**Published:** 2025-06-16

**Authors:** Sheila Castro-Suarez, Jonathan A. Zegarra-Valdivia, María Meza-Vega, Erik A. Guevara-Silva

**Affiliations:** 1 Basic Research Center on Dementia and Demyelinating Diseases of the Nervous System, National Institute of Neurological Sciences, Lima, Peru. Basic Research Center on Dementia and Demyelinating Diseases of the Nervous System National Institute of Neurological Sciences Lima Peru; 2 Global Brain Health Institute, San Francisco, California, United States. Global Brain Health Institute San Francisco California Estados Unidos; 3 Señor de Sipán University, Chiclayo, Peru. Señor de Sipán University Señor de Sipán University Chiclayo Peru; 4 Universidad Nacional Mayor de San Marcos, Lima, Perú. Universidad Nacional Mayor de San Marcos Universidad Nacional Mayor de San Marcos Lima Peru

**Keywords:** Dementia, Neurocognitive Disorders, Alzheimer Disease, Alzheimer Disease, Early Onset, Alzheimer Type Dementia

## Abstract

Early-onset Alzheimer’s disease (EOAD) accounts for between 5 and 10% of all cases of Alzheimer’s disease and is a rare and devastating form of the disease. This retrospective study analyzed the medical records of patients diagnosed with EOAD between 2022 and 2023 at a tertiary neurological center in Lima, Peru. Of 547 cases of dementia, 60 met the criteria for EOAD. Most were women (73.3%), and 71% had more than six years of education. The mean MMSE score was 11.92 ± 7.5, and the mean CDR score was 2, indicating severe cognitive impairment and moderate dementia. The amnestic variant was the most common clinical form, highlighting the involvement of episodic memory and executive function. The most common psychological and behavioral symptoms were depression and irritability. Atypical features such as myoclonus (5%) and epilepsy (10%) were also identified. These findings highlight the importance of healthcare professionals recognizing dementia in young adults at an early stage and offering comprehensive management to improve the quality of life of patients and their families.

## INTRODUCTION

Alzheimer’s disease (AD), the leading cause of dementia, begins with neuropathological changes in the brain in asymptomatic individuals and then progresses to clinical manifestations ^1^. It is classified into two types: early-onset AD (EOAD) and late-onset AD (LOAD) ^2^. Most cases are LOAD, while only 5-10% are EOAD (under 65 years of age). Within EOAD, 10% are familial, with autosomal dominant inheritance ^3^.

EOAD shows great variability in its clinical characteristics. Although the amnesic phenotype is the most common, as in LOAD, 25% of cases have non-amnesic (atypical) syndromes ^4^. EOAD most commonly affects language, visuospatial skills, executive functions, motor skills, and behavior ^5^. Cognitive symptoms are often accompanied by psychological and behavioral symptoms (PBS), which are more common in EOAD than in LOAD ^6^. In addition, EOAD has a more rapid and aggressive progression, with misdiagnosis or delayed diagnosis hindering its management and leading to psychosocial problems ^2^.

In Peru, as in other middle- and low-income countries, timely diagnosis of AD faces significant challenges. This study was conducted at a national referral neurological center in Peru with the aim of describing the clinical characteristics of patients with EOAD and comparing them according to sex.

**KEY MESSAGES**

Motivation for the study. To describe the clinical characteristics of early-onset Alzheimer’s disease (EOAD) and compare them according to gender. This condition is considered a rare disease, whose manifestations are still poorly understood.

Main findings. The most common clinical presentation of EOAD is the amnestic variant, which mainly affects episodic memory and executive function and is often accompanied by neuropsychiatric symptoms such as depression and irritability. Women tend to have more impairments in calculus, constructive apraxia, and visuospatial functions than men.

Implications for public health. By identifying the clinical characteristics of EOAD, healthcare professionals can recognize patients early on. Furthermore, it is essential to broaden the concept of dementia, avoiding limiting it exclusively to the population over 65 years of age.

## THE STUDY

### Study design

Descriptive, retrospective study, in which we reviewed the medical records of patients diagnosed with EOAD at the National Institute of Neurological Sciences (INCN) during the years 2022-2023. This institution is a third-level neurological center and a national referral center. In 2023, it provided approximately 68,000 consultations to patients with neurological disorders referred from a many different sociodemographic areas of Peru ^7^.

### Population

We reviewed 547 medical records with a diagnosis of dementia. Of these, 73 corresponded to early-onset dementia, but only 60 to EOAD in both its amnestic and non-amnestic phenotypes. The clinical records of EOAD met the diagnostic criteria for probable AD proposed by the National Institute on Aging-Alzheimer’s Association ^8^ and the criteria for non-amnesic phenotypes ^9^^-^^11^. All patients underwent brain MRI that showed the pattern of AD atrophy.

### Variables

The following variables were considered: sex, age at disease onset, years of education, time to diagnosis, history of neurodegenerative dementia, comorbidity, cognitive symptoms, psychological and behavioral symptoms, atypical neurological symptoms, EOAD phenotype, and functional assessment of dementia (Supplementary Table 1).

**Table 1:** Sociodemographic characteristics and performance on the mini-mental cognitive test and clinical dementia scale of patients with early-onset Alzheimer’s disease, according to sex.

Variables	Total (N=60)		Women (n=43)		Men (n=17)	
	Mean	SD	Mean	SD	Mean	SD
Age at diagnosis	59.1	6.7	60.3	5.7	56.4	8.3
Age at first symptom	55.0	6.7	56.2	5.8	52.1	8.1
Time to diagnosis	4.0	4.0	4.02	3.3	4.02	2.2
Years of education	9.9	4.6	9.2	4.6	11.8	4.0
Clinical variables						
MMSE	11.9	7.6	11.9	7.3	11.9	8.4
CDR	2		2		2	
History of neurodegenerative dementia (%)						
None	70		76.7		52.9	
Parkinson’s disease	3.3		4.7		0.0	
Alzheimer’s disease	23.3		18.6		35.3	
Dementia	3.3		0.0		11.8	

SD: standard deviation, MMSE: mini-mental state examination, CDR: clinical dementia rating scale

### Instruments

Tests such as the Wechsler Adult Intelligence Scale (WAIS-IV), Trail Making Test (A and B), Rey Figure Copying Test, Rey Auditory Verbal Learning Test, Wechsler Memory Scale, and Luria's Gnosis-Praxia assessment were used in 53% of the cases. All participants underwent the Mini-Mental State Examination (MMSE), with a maximum score of 30 points ^12^, and the Clinical Dementia Rating Scale (CDR), which classifies functional status as CDR 0 (no dementia), CDR 0.5 (mild cognitive impairment), CDR 1 (mild dementia), CDR 2 (moderate dementia), and CDR 3 (severe dementia) ^13^.

### Statistical analysis

Descriptive statistics, including frequencies and percentages, as well as measures of central tendency and dispersion (mean and standard deviation [SD] in variables with normal distribution) were used. Since not all variables were normally distributed, Spearman’s correlation was used to analyze the relationship between variables. In addition, preliminary comparisons were made with 95% confidence intervals corrected for bias using bootstrap resampling with 1000 repetitions (to correct for non-normality of the data). The analysis was performed using SPSS (v. 24.0., IBM).

### Ethical considerations

The study was reviewed and approved by the Institutional Committee on Research Ethics of the INCN (approval certificate No. 024-2022-CIEI-INCN).

## RESULTS

During the years 2022-2023, 547 medical records with a diagnosis of dementia were documented. Of these, 73 were diagnosed with early-onset dementia, and 13 were excluded because they were incomplete or corresponded to frontotemporal dementia. Finally, 60 medical records with a diagnosis of EOAD were included, of which 52 corresponded to the amnestic variant and 8 to the non-amnestic variant of EOAD.

We found that 72% (43) were women, and the average age at the first symptom and at diagnosis was lower in males than in females (52.1 SD: 8.1 and 56.2 SD: 5.8 years, respectively); with regard to years of education, 72% of cases had more than six years, but the mean for men was 11.8 (SD: 4) years. The time to diagnosis of EOAD was 4.1 (SD: 2.9) years. On the scales applied, the mean MMSE score was 11.9 (SD: 7.6), similar between men and women, and the CDR score was 2, indicating a moderate degree of dementia. On the other hand, the distribution of family history of AD and dementia (first-degree relatives) was 47.1% in men (Table 1).

Episodic memory impairment was the most prevalent cognitive symptom (100%), followed by executive dysfunction (98.3%), while acalculia was found in 88.3%. Other significant deficits included constructive apraxia (76.7%), language impairments (comprehension deficit 70.0%), and visuospatial impairment (68.3%). Among psychological and behavioral symptoms (PBS), depression was the most frequent symptom (53.3%), followed by irritability and anxiety (51.7% and 41.7%, respectively). Other clinical manifestations (atypical) were reported: myoclonus (5%) and epilepsy (10%) (Table 2).

**Table 2:** Specific clinical characteristics of patients with early-onset Alzheimer's disease, by sex.

Clinical features		Total (N=60)	Women (n=43)	Men (n=17)
		N (%)	n (%)	n (%)
Cognitive symptoms				
	Episodic memory impairment	60 (100.0)	43(100.0)	17(100.0)
	Executive dysfunction	59 (98.3)	42 (97.7)	17 (100.0)
	Acalculia	53 (88.3)	39 (90.7)	14 (82.4)
	Constructive apraxia	46 (76.7)	34 (79.1)	12 (70.6)
	Comprehension impairment	42 (70.0)	33 (76.7)	9 (52.9)
	Visual-spatial impairment	41 (68.3)	33 (76.7)	8 (47.1)
	Impairment in phrase repetition	34 (56.7)	25 (58.1)	9 (52.9)
	Anomia	29 (48.3)	22 (51.2)	7 (41.2)
	Agraphia	22 (36.7)	18 (41.9)	4 (23.5)
	Word recall impairment	20 (33.3)	15 (34.9)	5 (29.4)
	Object knowledge Impairment	19 (31.7)	14 (32.6)	5 (29.4)
	Dressing apraxia	18 (30.0)	11 (25.6)	7 (41.2)
	Alexia	18 (30.0)	12 (27.9)	6 (35.3)
	Left/right disorientation	17 (28.3)	12 (27.9)	5 (29.4)
	Agnosia	15 (25.0)	10 (23.3)	5 (29.4)
	Word repetition impairment	14 (23.3)	9 (20.9)	5 (29.4)
	Paraphasia	13 (21.7)	9 (20.9)	4 (23.5)
	Object perception impairment	9 (15.0)	6 (14.0)	3 (17.6)
Psychological and behavioral symptoms				
	Depression	32 (53.3)	22 (51.2)	10 (58.8)
	Irritability	31 (51.7)	22 (51.2)	9 (52.9)
	Anxiety	25 (41.7)	20 (46.5)	5 (29.4)
	Delusional ideas	22 (36.7)	16 (37.2)	6 (35.3)
	Abnormal motor behavior	21 (35)	16 (37.2)	5 (29.4)
	Apathy	21 (35)	14 (32.6)	7 (41.2)
	Hallucinations	17 (28.3)	14 (32.6)	3 (17.6)
	Nighttime behavior impairment	10 (16.7)	8 (18.6)	2 (11.8)
	Aggression/Agitation	9 (15.0)	7 (16.3)	2 (11.8)
	Appetite disorders	9 (15.0)	5 (11.6)	4 (23.5)
	Disinhibition	6 (10.0)	4 (9.3)	2 (11.8)
	Lack of empathy	4 (6.7)	4 (9.3)	0 (0.0)
	Euphoria	2 (3.3)	2 (4.7)	0 (0.0)
Atypical neurological symptoms				
	Epilepsy	6 (10)	2 (4.7)	4 (23.5)
	Myoclonus	3 (5.0)	3 (7.0)	0 (0.0)

The analysis of the clinical characteristics of patients with EOAD according to sex showed that women had higher frequency of acalculia (90.7%) and constructive apraxia (79.1%) compared to men (82.4% and 70.6%). Meanwhile, men had higher rates of dressing apraxia (41.2%) and alexia (35.3%) compared to women (25.6% and 27.9%). When analyzing PBS, we found that depression and anxiety were more frequent in men (58.8% and 52.9%), while anxiety, delusions, and aberrant motor behavior were more frequent in women (46.5%, 37.2%, and 37.2%, respectively) compared to men. Among atypical neurological symptoms, myoclonus was present in 7% of women and epilepsy in 23.5% of men (Table 2).

More than half of the cases with EOAD presented the disease in isolation, without additional clinical comorbidities. Specifically, 53.6% of women (23 out of 43) and 64.7% of men (11 out of 17) had no diagnoses other than EOAD. The most common comorbidities in both sexes were prediabetes/type 2 diabetes mellitus (10%, 6 cases), high blood pressure (8.3%, 5 cases), and vitamin B12 deficiency (8.3%, 5 cases). Some conditions were more frequent in women: prediabetes/diabetes (11.6%, 5 cases), dyslipidemia (9.3%, 4 cases), and high blood pressure (9.3%, 4 cases); while these conditions were less common in men, with only one case of prediabetes/diabetes (5.9%), no cases of dyslipidemia, and one case of high blood pressure (5.9%). On the other hand, mood disorders, such as anxiety and depression, were only present in men (11.8%, 2 cases).

**Figure 1:**
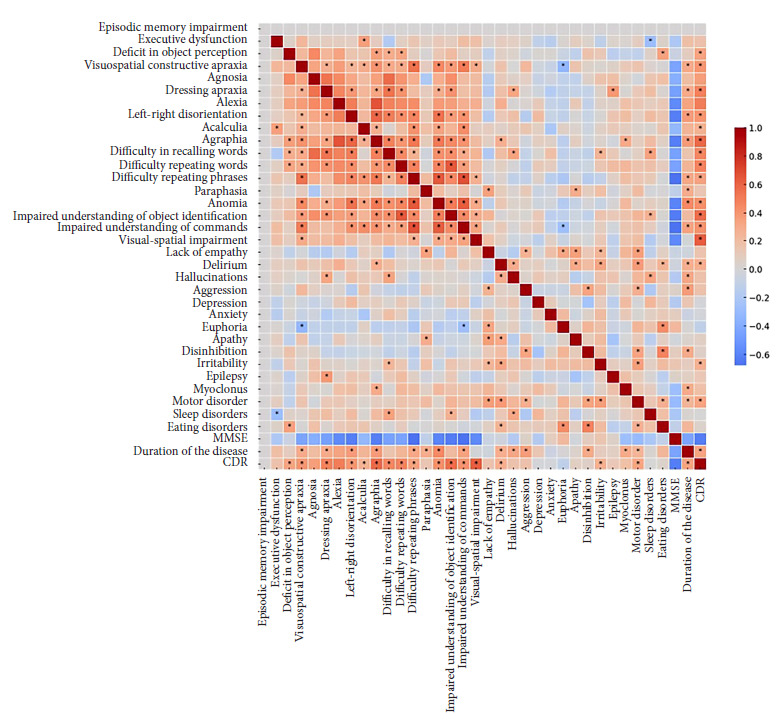
Spearman correlation matrix between clinical manifestations, performance on the mini-mental cognitive test, clinical dementia scale, and time to diagnosis.

Figure 1 shows a heat map of correlations between cognitive variables, PBS, and atypical neurological variables in EOAD (p<0.05). We explored the correlations between the variables and observed a positive correlation between executive dysfunction and acalculia, and between the latter and other deficits such as visuospatial constructive apraxia, agnosia, word repetition, and anomia, implying the involvement of posterior brain areas. Constructive apraxia, agnosia, and dressing apraxia are also strongly correlated with each other, suggesting that these visuospatial and motor difficulties are common in the progression of EOAD. Within PBS, irritability, aggression, and disinhibition are positively correlated, as are depression and anxiety. Hallucinations and delusions are correlated with each other, and both correlate with the severity of dementia (CDR: 2).

## DISCUSSION

The study describes the clinical characteristics of EOAD in a national referral center in Peru. The most common presentation was the amnesic variant, accompanied by classic neuropsychiatric symptoms. Women showed greater impairment in calculus, constructive apraxia, and visuospatial function than men. Regarding PBS, women reported more anxiety and men reported more depressive symptoms.

The average age at onset of symptoms was 55 years and most patients with EOAD had severe cognitive impairment in moderate stages of dementia. This contrasts with the findings of Altomari *et al*. (Italy) and Apostolova *et al*. (USA), in which the average age was 58 and 60 years, respectively, and patients were in mild stages (CDR: 0.5/1 and 0.5); MMSE scores were 16 and 22 ^6^^,^^14^. Higher MMSE scores: 17 have been reported in Peru in cases of LOAD with CDR: 2 ^15^. Our patients presented symptoms 3-5 years earlier, a finding similar to that from a Latin American population in which the time to onset of symptoms was 6.8 years ^16^. Higher MMSE scores in non-Latino populations with EOAD and in Peruvian LOAD reflect, on the one hand, that diagnoses are late in our population and, on the other hand, that EOAD is more severe compared to LOAD. This shows the influence of social health determinants (education, access to medical care, economic conditions, etc.) in low- and middle-income countries, as reported in a Peruvian study where social determinants increase the risk of AD ^17^.

The most affected cognitive domains in this study were episodic memory and executive function. These findings contrast with those reported by Tellechea and Echevaste ^5^^,^^18^, who point out that in early stages, memory is relatively well preserved, with alterations in attention, executive function, and visuospatial skills being more frequent. In our study, most participants were in the moderate stage of dementia, which could explain the greater impact on memory. In terms of PBS, depression, irritability, and anxiety were found more frequently, unlike the Italian longitudinal study, in which apathy (62.5%), agitation (49.6%), and depression (49.4%) predominated ^6^. This difference may be due to the study design, as the Italian study was prospective and used the Neuropsychiatric Inventory (NPI) for recording, while ours was retrospective, and symptoms may not have been fully recorded due to limited consultation time. On the other hand, PBS (depression and irritability) were present in more than 50% of our cases, as reported in the study of EOAD compared to LOAD in Italy ^6^, while a study in China found higher NPI scores in LOAD ^19^. Despite these diverse findings, it is important to note that early identification and appropriate management of PBS can improve the quality of life of the patient and caregiver. In addition, the positive correlations between irritability, disinhibition, and aggression underscore the need for timely intervention to prevent caregiver overload.

When analyzing the frequency of cognitive symptoms by sex, in addition to memory and executive function impairment, we found that acalculia and constructive apraxia were more frequent in women. This impairment coincides with the findings by Contador *et al*. in Spanish patients, who found that women with EOAD had greater deterioration and brain atrophy than men ^20^. On the other hand, magnetic resonance imaging studies show more marked parietal atrophy in EOAD compared to LOAD ^21^. These findings support a clinical-anatomical correlation in the affected temporo-parietal regions and suggest a possible influence of sex on the spread and susceptibility to EOAD.

The main limitation of the study is its retrospective design, with records of variable quality; in addition, only half of the cases underwent neuropsychological assessment. Furthermore, vitamin B12 deficiency and hypothyroidism were diagnosed as comorbidities in less than 10% of women, which could influence the cognitive status of the cases. However, a Peruvian study found no association between metabolic and endocrine disorders and cognitive impairment ^22^. Nevertheless, as this is a rare disease, having 60 records with a diagnosis of EOAD in a national referral institution provides valuable information that is relevant in view of the growing global burden of AD and the need for timely diagnosis in resource-limited settings. We recommend conducting prospective studies that allow for better control of variables and biases. Among the variables, we suggest considering social determinants of health that could influence delayed diagnosis.

In conclusion, the amnestic variant was the most common form of EOAD, and mostly affected episodic memory and executive function. The most frequent PBS were anxiety, depression, and irritability. Most cases were in the moderate stage of dementia. These findings highlight the importance of healthcare professionals recognizing dementia in young adults at an early stage and offering comprehensive management to improve the quality of life of patients and their families.
