# Supplementary material for: Clinical profile of early-onset Alzheimer’s disease in Peru: case series from a neurological care center
Source: Rev Peru Med Exp Salud Publica. 2025 Jun 16;42(2):196–202. doi: 10.17843/rpmesp.2025.422.14413 (PMC12377887; doi:10.17843/rpmesp.2025.422.14413)
Supplement: Translation

# Translation: Perfil clínico de la enfermedad de Alzheimer de inicio precoz en el Perú: serie de casos de un centro de atención neurológica

**Authors:** Sheila Castro-Suarez, Jonathan A. Zegarra-Valdivia, María Meza-Vega, Erik A. Guevara-Silva

**Affiliations:** 1 Centro Básico de Investigación en Demencias y Enfermedades Desmielinizantes del Sistema Nervioso, Instituto Nacional de Ciencias Neurológicas, Lima, Perú.; 2 Global Brain Health Institute, San Francisco, California, Estados Unidos.; 3 Universidad Señor de Sipán, Chiclayo, Perú.; 4 Universidad Nacional Mayor de San Marcos, Lima, Perú.

**Keywords:** Demencia, Desordenes neurocognitivos, Enfermedad de Alzheimer, Enfermedad de Alzheimer de inicio temprano, Demencia tipo Alzheimer

## Abstract

La enfermedad de Alzheimer de inicio precoz (EAIP) representa entre el 5 y 10% de todos los casos de enfermedad de Alzheimer, constituye una forma rara y devastadora de esta condición. Este estudio retrospectivo analizó las historias clínicas de pacientes diagnosticados con EAIP entre 2022 y 2023 en un centro neurológico terciario en Lima, Perú. De 547 casos de demencia, 60 cumplían criterios para EAIP. La mayoría fueron mujeres (73,3%), el 71% tenía más de seis años de educación. La media del MMSE fue de 11,92 ± 7,5 y la del CDR fue de 2, indicando deterioro cognitivo severo y demencia moderada. La variante amnésica fue la forma clínica más común, destacando el compromiso de la memoria episódica y la función ejecutiva. Los síntomas psicológicos y conductuales más frecuentes fueron la depresión y la irritabilidad. También se identificaron características atípicas como mioclonías (5%) y epilepsia (10%). Estos hallazgos destacan la importancia de que los profesionales de salud reconozcan la demencia en adultos jóvenes, en fases tempranas y ofrezcan un manejo integral que mejore la calidad de vida de los pacientes y sus familias.

## INTRODUCCIÓN

La enfermedad de Alzheimer (EA), principal causa de demencia comienza con cambios neuropatológicos cerebrales en personas asintomáticas, y luego progresa hasta presentar manifestaciones clínicas ^1^. Se clasifica en dos tipos: de inicio precoz o temprano (EAIP) y de inicio tardío (EAIT) ^2^. La mayoría de los casos son EAIT, mientras que solo el 5-10% corresponde a EAIP (menores de 65 años). Dentro de la EAIP, el 10% es de tipo familiar, con herencia autosómica dominante ^3^.

La EAIP muestra una gran variabilidad en sus características clínicas. Aunque el fenotipo amnésico es el más común, como en la EAIT, el 25% de los casos presentan síndromes no amnésicos (atípicos) ^4^. La EAIP afecta con mayor frecuencia el lenguaje, las habilidades visuoespaciales, las funciones ejecutivas, las habilidades motoras y el comportamiento ^5^. Los síntomas cognitivos suelen ir acompañados de síntomas psicológicos y conductuales (SPC), que son más comunes en la EAIP que en la EAIT ^6^. Además, la EAIP presenta una progresión más rápida y agresiva, con diagnósticos erróneos o tardíos que dificultan su manejo y generan problemas psicosociales ^2^.

En el Perú, al igual que en países de ingresos medios y bajos, el diagnóstico oportuno de la EA enfrenta importantes desafíos. El presente estudio se realizó en un centro neurológico de referencia nacional de Perú, con el objetivo de describir las características clínicas de los pacientes con EAIP y compararlos según sexo.

**MENSAJE CLAVE**

Motivación para realizar el estudio. Describir las características clínicas de la enfermedad de Alzheimer de inicio precoz (EAIP) y compararlo según sexo. Esta entidad es considerada una enfermedad rara, cuyas manifestaciones aún son poco conocidas.

Principales hallazgos. La presentación clínica más común de la EAIP es la variante amnésica, que afecta principalmente la memoria episódica y la función ejecutiva, y a menudo se acompaña de síntomas neuropsiquiátricos como depresión e irritabilidad. Las mujeres tienden a presentar más alteraciones en el cálculo, apraxia constructiva y funciones visuoespaciales que los hombres.

Implicancias para la salud pública. Al identificar las características clínicas del EAIP, los profesionales de la salud pueden reconocer a los pacientes de manera temprana. Por otro lado, es fundamental ampliar la concepción de la demencia, evitando limitarla exclusivamente a la población mayor de 65 años.

## EL ESTUDIO

### Diseño del estudio

Estudio descriptivo, retrospectivo; se revisaron las historias clínicas de pacientes diagnosticados con EAIP en el Instituto Nacional de Ciencias Neurológicas (INCN) durante los años 2022-2023. Esta institución es un centro neurológico de tercer nivel, de referencia nacional. En el 2023 brindo alrededor de 68 000 atenciones a pacientes con trastornos neurológicos, referidos desde una amplia área sociodemográfica del Perú ^7^.

### Población

Se revisaron un total de 547 historias clínicas con diagnóstico de demencia. De estas, 73 correspondieron a demencia de inicio temprano, pero solo 60 a EAIP tanto en su fenotipo amnésico como no amnésico. Los registros clínicos de EAIP cumplieron con los criterios diagnósticos de EA probable propuestos por la *National Institute on Aging-Alzheimer’s Association*^8^ y los criterios para los fenotipos no amnésicos ^9^^-^^11^. Todos tuvieron una resonancia magnética de encéfalo que mostró el patrón de atrofia de la EA.

### Variables

Se consideraron al sexo, edad de inicio de enfermedad, años de educación, tiempo al diagnóstico, antecedente de demencia neurodegenerativa, comorbilidad, síntoma cognitivo, síntoma psicológicos y conductuales, síntomas neurológicos atípicos, fenotipo de EAIP y valoración funcional de la demencia (tabla suplementaria 1).

### Instrumentos

El 53% de los casos fue evaluado con pruebas como la Escala de Inteligencia para Adultos de Wechsler (WAIS-IV), Test del Trazo (A y B), Test de Copia de la Figura de Rey, Test de Aprendizaje Verbal Auditivo de Rey, Escala de Memoria de Wechsler y evaluación de Gnosia-Praxia de Luria. En todos los participantes se consignó el examen cognoscitivo mini-mental (MMSE de sus siglas en inglés), con una puntuación máxima de 30 puntos ^12^, y la Escala Clínica de Demencia (CDR de sus siglas en inglés), que clasifica el estado funcional en CDR 0 (sin demencia), CDR 0.5 (deterioro cognitivo leve), CDR 1 (demencia leve), CDR 2 (demencia moderada) y CDR 3 (demencia severa) ^13^.

### Análisis estadístico

Se utilizaron estadísticas descriptivas, incluyendo frecuencias y porcentajes, así como medidas de tendencia central y dispersión (media y desviación estándar [DE] en las variables con distribución normal). Debido a que no todas las variables presentaron distribución normal, se utilizó la correlación de Spearman para analizar la relación entre variables. Además, se realizaron comparaciones previas con intervalos de confianza del 95% corregidos por sesgo mediante remuestreo por bootstrap con 1000 repeticiones (para corregir la no normalidad de los datos). El análisis se realizó en el programa SPSS (v. 24.0., IBM).

### Consideraciones éticas

El estudio fue revisado y aprobado por el Comité Institucional de Ética en Investigación del INCN (certificado de aprobación Nº 024-2022-CIEI-INCN).

## RESULTADOS

Durante los años 2022-2023, se documentaron 547 historias clínicas con diagnóstico de demencia. De estas, 73 fueron identificadas con un diagnóstico de demencia de inicio temprano, y 13 fueron excluidas por estar incompletas o corresponder a demencia frontotemporal. Finalmente, se incluyeron 60 historias clínicas con diagnóstico de EAIP, de las cuales 52 correspondían a la variante amnésica y 8 a la variante no amnésica de EAIP.

El 72% (43) fueron mujeres, la media de la edad al primer síntoma y al diagnóstico fue menor en varones que en mujeres (52,1 DE: 8,1 y 56,2 DE: 5,8 años, respectivamente); respecto a los años de educación, el 72% de los casos tuvo más de seis años, pero la media en varones fue de 11,8 (DE: 4) años. El tiempo al diagnóstico de EIAP fue de 4,1 (DE: 2,9) años. En las escalas aplicadas, la puntuación media en el MMSE fue de 11,9 (DE: 7,6), similar entre hombres y mujeres, y el puntaje del CDR fue de 2, indicando un grado moderado de demencia. Por otro lado, la distribución del antecedente familiar de EA y demencia (familiares de primer grado) fue del 47,1% en varones (tabla 1).

**Tabla 1:** Características sociodemográficas y rendimiento en la escala del examen cognoscitivo mini-mental y la escala clínica de demencia de los pacientes con enfermedad de Alzheimer de inicio precoz, según sexo.

Variables	Total (N=60)		Femenino (n=43)		Masculino (n=17)	
	Media	DE	Media	DE	Media	DE
Edad al diagnóstico	59,1	6,7	60,3	5,7	56,4	8,3
Edad al primer síntoma	55,0	6,7	56,2	5,8	52,1	8,1
Tiempo al diagnóstico	4,0	4,0	4.02	3,3	4,02	2,2
Años de educación	9,9	4,6	9,2	4,6	11,8	4,0
Variables clínicas						
MMSE	11,9	7,6	11,9	7,3	11,9	8,4
CDR	2		2		2	
Antecedente de demencia neurodegenerativa (%)						
Ninguno	70		76,7		52,9	
Enfermedad de Parkinson	3,3		4,7		0,0	
Enfermedad de Alzheimer	23,3		18,6		35,3	
Demencia	3,3		0,0		11,8	

DE: desviación estándar, MMSE: examen cognoscitivo mini-mental, CDR: escala clínica de demencia

Dentro de los síntomas cognitivos, el deterioro de la memoria episódica fue el síntoma predominante (100%), seguido por la disfunción ejecutiva (98,3%), mientras la acalculia estuvo presente en el 88,3%. Otros déficits significativos incluyen apraxia constructiva (76,7%), alteraciones en el lenguaje (déficit en la comprensión 70,0%) y alteración visuoespacial (68,3%). Dentro de los síntomas psicológicos y conductuales (SPC) la depresión fue el síntoma más frecuente 53,3%, seguida por la irritabilidad y la ansiedad (51,7 y 41,7%, respectivamente). Se reportaron otras manifestaciones clínicas (atípicas): mioclonías 5 % y epilepsia 10% (tabla 2).

**Tabla 2:** Características clínicas específicas de los pacientes con enfermedad de Alzheimer de inicio precoz, según sexo.

Características clínicas		Total (N=60)	Femenino (n=43)	Masculino (n=17)
		N (%)	n (%)	n (%)
Síntomas cognitivos				
	Deterioro de la memoria episódica	60 (100,0)	43(100,0)	17(100,0)
	Disfunción ejecutiva	59 (98,3)	42 (97,7)	17 (100,0)
	Acalculia	53 (88,3)	39 (90,7)	14 (82,4)
	Apraxia constructiva	46 (76,7)	34 (79,1)	12 (70,6)
	Deterioro en la comprensión	42 (70,0)	33 (76,7)	9 (52,9)
	Alteración visuoespacial	41 (68,3)	33 (76,7)	8 (47,1)
	Deterioro en la repetición de frases	34 (56,7)	25 (58,1)	9 (52,9)
	Anomia	29 (48,3)	22 (51,2)	7 (41,2)
	Agrafia	22 (36,7)	18 (41,9)	4 (23,5)
	Deterioro en la recuperación de una palabra	20 (33,3)	15 (34,9)	5 (29,4)
	Deterioro en el conocimiento de objetos	19 (31,7)	14 (32,6)	5 (29,4)
	Apraxia del vestir	18 (30,0)	11 (25,6)	7 (41,2)
	Alexia	18 (30,0)	12 (27,9)	6 (35,3)
	Desorientación izquierda/derecha	17 (28,3)	12 (27,9)	5 (29,4)
	Agnosia	15 (25,0)	10 (23,3)	5 (29,4)
	Deterioro en la repetición de palabras	14 (23,3)	9 (20,9)	5 (29,4)
	Parafasias	13 (21,7)	9 (20,9)	4 (23,5)
	Déficit en la percepción de objetos	9 (15,0)	6 (14,0)	3 (17,6)
Síntomas psicológicos y conductuales				
	Depresión	32 (53,3)	22 (51,2)	10 (58,8)
	Irritabilidad	31 (51,7)	22 (51,2)	9 (52,9)
	Ansiedad	25 (41,7)	20 (46,5)	5 (29,4)
	Ideas delirantes	22 (36,7)	16 (37,2)	6 (35,3)
	Comportamiento motor aberrante	21 (35)	16 (37,2)	5 (29,4)
	Apatía	21 (35)	14 (32,6)	7 (41,2)
	Alucinaciones	17 (28,3)	14 (32,6)	3 (17,6)
	Alteraciones del comportamiento nocturno	10 (16,7)	8 (18,6)	2 (11,8)
	Agresividad/Agitación	9 (15,0)	7 (16,3)	2 (11,8)
	Trastornos del apetito	9 (15,0)	5 (11,6)	4 (23,5)
	Desinhibición	6 (10,0)	4 (9,3)	2 (11,8)
	Falta de empatía	4 (6,7)	4 (9,3)	0 (0,0)
	Euforia	2 (3,3)	2 (4,7)	0 (0,0)
Síntomas neurológicos atípicos				
	Epilepsia	6 (10)	2 (4,7)	4 (23,5)
	Mioclonías	3 (5,0)	3 (7,0)	0 (0,0)

En el análisis de las características clínicas de pacientes con EAIP según el sexo, en los síntomas cognitivos las mujeres mostraron mayor frecuencia de acalculia (90,7%) y apraxia constructiva (79,1%) frente a los varones (82,4% y 70,6%). Mientras los varones presentaron mayor apraxia del vestir (41,2%) y alexia (35,3%) en comparación con las mujeres (25,6% y 27,9%). En el análisis de los SPC, la depresión y la ansiedad fueron más frecuentes en varones (58,8 y 52,9%), mientras que la ansiedad, las ideas delirantes y el comportamiento motor aberrante más frecuentes en mujeres (46,5%, 37,2% y 37,2% respectivamente) comparado con los varones. Dentro de los síntomas neurológicos atípicos la mioclonía se presentó en el 7%, de mujeres y la epilepsia en un 23,5% de varones (tabla 2).

Más de la mitad de los casos con EAIP presentaron la enfermedad de forma aislada, sin comorbilidades clínicas adicionales. Específicamente, el 53,6 % de las mujeres (23 de 43) y el 64,7 % de los varones (11 de 17) no tenían diagnósticos asociados distintos a EAIP. Las comorbilidades más frecuentes en ambos sexos fueron la prediabetes/diabetes mellitus tipo 2 (10%, 6 casos), la hipertensión arterial (8,3%, 5 casos) y la deficiencia de vitamina B12 (8,3%, 5 casos). Algunas condiciones se observaron con mayor frecuencia en mujeres: prediabetes/diabetes (11,6%, 5 casos), dislipidemia (9,3%, 4 casos) e hipertensión arterial (9,3%, 4 casos); mientras que en varones estas condiciones fueron menos comunes, registrándose un solo caso de prediabetes/diabetes (5,9%), ningún caso de dislipidemia y un caso de hipertensión arterial (5,9%). Por otro lado, los trastornos del estado de ánimo, como ansiedad y depresión, se presentaron únicamente en varones (11,8%, 2 casos).

La figura 1 presenta un mapa de calor de correlaciones entre variables cognitivas, SPC y neurológicas atípicas en EAIP (p<0,05). Exploramos la correlaciones entre las variables y observamos una correlación positiva entre disfunción ejecutiva y acalculia, y de esta última con otros déficits como apraxia constructiva visuoespacial, agnosia, repetición de palabras y anomia, lo que implica la participación de áreas cerebrales posteriores. También se correlacionan fuertemente entre sí la apraxia constructiva, la agnosia y la apraxia del vestido, lo que sugiere que estas dificultades visuoespaciales y motoras son comunes en la progresión del EAIP. Dentro de los SPC la irritabilidad, agresividad y desinhibición están positivamente correlacionadas, al igual que la depresión y la ansiedad. Las alucinaciones y delirios se correlacionan entre sí, y ambas correlacionan con la severidad de la demencia (CDR: 2).

**Figura 1:** Matriz de correlación de Spearman entre las manifestaciones clínicas, el desempeño en el exa-men cognoscitivo mini-mental, escala clínica de demencia y el tiempo al diagnóstico.

## DISCUSIÓN

El estudio describe las características clínicas del EAIP en un centro de referencia nacional de Perú. La presentación más frecuente fue la variante amnésica, acompañada de síntomas neuropsiquiátricos clásicos. Las mujeres mostraron mayor deterioro en cálculo, apraxia constructiva y función visuoespacial que los hombres. En cuanto a los SPC, las mujeres reportaron más ansiedad y los hombres más síntomas depresivos.

La edad media al primer síntoma fue de 55 años, la mayoría de los pacientes con EAIP presentaba deterioro cognitivo severo en etapas moderadas de demencia. Esto contrasta con los hallazgos de Altomari *et al*. (Italia) y Apostolova *et al*. (EE. UU.), donde la edad promedio fue de 58 y 60 años, respectivamente; y se encontraban en etapas leves (CDR: 0,5/1 y 0,5); las puntuaciones MMSE en 16 y 22 ^6^^,^^14^. Puntuaciones mayores de MMSE: 17 se han reportado en Perú en casos de EAIT con CDR: 2 ^15^. Nuestros pacientes presentaron síntomas 3-5 años antes, hallazgo similar a lo encontrado en una población latina donde el inicio de síntomas fue 6,8 años ^16^. Puntuaciones mayores de MMSE en poblaciones no latinas con EAIP y en peruanos EAIT refleja por un lado, que los diagnósticos son tardíos en nuestra población y, por otro, que la EAIP es más severa comparada con la EAIT. Esto muestra la influencia de los determinantes sociales de la salud (educación, acceso a la atención médica, condiciones económicas, etc) en países de ingresos bajos y medianos, como lo reportado en un estudio peruano donde los determinantes sociales aumentan el riesgo de EA ^17^.

En el presente estudio, los dominios cognitivos más afectados fueron la memoria episódica y la función ejecutiva. Estos hallazgos contrastan con lo reportado por Tellechea y Echevaste ^5^^,^^18^, quienes señalan que en etapas tempranas la memoria se conserva relativamente bien, siendo más frecuentes las alteraciones en atención, función ejecutiva y habilidades visuoespaciales. En nuestro estudio, la mayoría se encontraba en etapa moderada de demencia, lo que podría explicar la mayor afectación de la memoria. En cuanto a los SPC, se encontraron con mayor frecuencia depresión, irritabilidad y ansiedad, a diferencia del estudio longitudinal italiano donde predominaban apatía (62,5%), agitación (49,6%) y depresión (49,4%) ^6^. Esta diferencia puede deberse al diseño del estudio, ya que el estudio italiano fue prospectivo y utilizó el Inventario Neuropsiquiátrico (INP) para el registro, mientras el nuestro fue retrospectivo, y los síntomas podrían no haberse registrado completamente debido al tiempo limitado en consulta. Por otro lado, los SPC (depresión e irritabilidad) estuvieron presentes en más del 50% de nuestros casos, como el reportado en el estudio de EAIP comparado a EAIT en Italia ^6^, mientras un estudio en China encontró puntuaciones del INP más altas en EAIT ^19^. A pesar de estos hallazgos diversos, se resalta que la identificación temprana y el manejo adecuado de los SPC pueden mejorar la calidad de vida del paciente y del cuidador. Además, las correlaciones positivas entre irritabilidad, desinhibición y agresividad subrayan la necesidad de intervenir a tiempo para evitar la sobrecarga del cuidador.

Al analizar la frecuencia de los síntomas cognitivos por sexo, además del compromiso en memoria y función ejecutiva, se encontró que la acalculia y la apraxia constructiva fueron más frecuentes en mujeres. Este compromiso coincide con lo reportado por Contador *et al*. en españoles, donde las mujeres con EAIP presentaron mayor deterioro y atrofia cerebral que los hombres ^20^. Por otro lado, estudios de resonancia magnética muestran una atrofia parietal más marcada en EAIP comparada con EAIT ^21^. Estos hallazgos respaldan un correlato clínico-anatómico en las regiones temporo-parietales afectadas y sugieren una posible influencia del sexo en la propagación y susceptibilidad a la EAIP.

La principal limitación del estudio es su diseño retrospectivo, con registros de calidad variable; además, solo la mitad de los casos contó con una evaluación neuropsicológica. Por otro lado, en menos del 10% de las mujeres la deficiencia de vitamina B12 y el hipotiroidismo fue diagnosticado como comorbilidad, que podría influir en el estado cognitivo de los casos. Sin embargo, un estudio peruano no encontró asociación entre los trastornos metabólicos y endocrinos; y el deterioro cognitivo ^22^. No obstante, al tratarse de una enfermedad rara, disponer de 60 registros con diagnóstico de EAIP en una institución de referencia nacional aporta información valiosa, relevante frente a la creciente carga global de EA y la necesidad de diagnósticos oportunos en contextos con recursos limitados. Recomendamos realizar estudios prospectivos que permitan un mejor control de variables y sesgos, dentro de las variables sugerimos considerar los determinantes sociales en salud que podrían influir en el retraso en el diagnóstico.

En conclusión, la variante amnésica fue la forma más común de EAIP, con afectación predominante de la memoria episódica y la función ejecutiva. Los SPC más frecuentes fueron la ansiedad, depresión e irritabilidad. La mayoría de casos se encontraba en estadio moderado de demencia. Estos hallazgos destacan la importancia de que los profesionales de salud reconozcan la demencia en adultos jóvenes, en fases tempranas y ofrezcan un manejo integral que mejore la calidad de vida de los pacientes y sus familias.
